# Diagnosis and Some of the Clinical Aspects of Gyroma and Endothelioma of the Ovary

**Published:** 1892-11

**Authors:** Mary A. Dixon Jones

**Affiliations:** 163 DeKalb Avenue; Brooklyn, N. Y.


Buffalo Medical and Surgical Journal

VOL. XXXII. NOVEMBER, 1892. NO. 4.

### Original Communications.

### DIAGNOSIS AND SOME OF THE CLINICAL ASPECTS OF GYROMA AND ENDOTHELIOMA OF THE OVARY.

BY MARY A. DIXON JONES, M. D., Brooklyn, N. Y.

ONLY recently has the attention of the medical profession been called to the disease known as "endothelioma of the ovary, changing to angioma and hematoma." While it has many features of great interest, we, as yet, do not understand its full import, or its grave ulterior consequences. The disease is sufficient to destroy the comfort, health and activities of the individual, and may even grow to conditions that will imperil and shorten life.

This growth is always proceeded by chronic inflammation or oöphoritis, by which process the anatomical elements of the ovary are more or less transformed into an embryonal medullary tissue. From this newly formed tissue are developed the endothelia, long branching protoplasmic bodies, filled with living matter in the shape of coarse granules and hematoblasts. These protoplasmic masses have a striking resemblance to net-celled sarcoma, and, like it, they have the power of changing into their own any and every tissue of the ovary. The endothelia are seen to be gradually transformed into blood-corpuscles and blood-vessels, and thus is formed between unchanged endothelia, a collection of blood and blood-vessels. The growth under consideration is, then, a profuse new formation of red-blood corpuscles and blood-vessels, mainly of capillary or venous nature, and at last terminates in what we know to be hematoma of the ovary. The ovary thus, in full development of the disease, becomes a blood cyst, and is usually found adherent to the adjacent organs in consequence of repeated attacks of peritonitis. As the hematoma grows and increases in bulk, a gradual thinning of the sac or capsule of the ovary takes place with the danger of rupture. Dr. H.

J. Boldt reported at the International Medical Congress, in Berlin, in 1890, a case where such a hematoma had ruptured, causing hemorrhage into the peritoneal cavity, and the patient's life was saved only by an immediate operation, and the removal of the ovary with the ruptured hematoma. Dr. Francis Foerster in his paper, Clinical and Microscopical Analysis of Twenty-five Extirpated Ovaries, mentions as the outcome of a ruptured hematoma a hemorrhage into the peritoneal cavity, or into the broad ligament.[1]

It has been stated by some authors that endothelioma of the ovary could not be diagnosticated before operation. This, if true, would be extremely unfortunate. I have studied this peculiar form of degeneration in over thirty cases, both the pathological changes in the affected organs, and the clinical condition of the patients, noting carefully the characteristics which invariably accompany a marked development of the disease. Its symptoms, as thus far understood, seem sufficiently clear and distinct to enable one to diagnosticate it with certainty. I stated in an article published in the *New York Medical Journal*, September 28, 1889:

> The clinical features in this disease are so pronounced that in many instances I have recognized it on first examination, and, in every case, when followed by an operation, my diagnosis was verified. The patients all complain of a characteristic pain in the region of the ovaries, at times severe, sharp and lancinating; they have a peculiar pale, cachectic look, or the cadaveric pallor of the consumptive, and a marked emaciation. Some who are naturally strong, with a tendency to embonpoint, from the time of the development of the disease begin to emaciate—in some instances lose twenty, thirty or forty pounds. The more advanced the disease. the more emaciated they become, and the more extreme the pallor. The more the ovary is filled with this growth, the more serious are the manifestations in the system, and the more the general health, comfort, and active usefulness of the individual are destroyed.

Without knowing the existence of the disease in the ovary, or understanding its nature, we still see in the patients indications and evidences of a profound and serious trouble, and such, as in most instances, would demand an immediate operation. At first I diagnosticated all these cases as chronic oöphoritis, accompanied by some obscure pathological changes; now I am able, in almost every case, to say positively when endothelioma exists.

The first patient, in whom I found this peculiar degeneration, was a single woman, nearly forty years of age, who consulted me

1. The *American Journal of Obstetrics*, May, 1892, page 598.

in June, 1885. So great was the constitutional disturbance, without a correspondingly serious derangement of any of the vital organs, that to me it was an enigma, and a matter of earnest consideration. Though the intense inflammation of the enlarged and prolapsed ovaries did, to some extent, explain the local pain, it did not seem sufficient to account for the patient's generally grave symptoms, her cachectic appearance, the peculiar pallor, the feebleness, the emaciation, or the extreme nervousness and hysteria; nor did it seem a sufficient cause for her disturbed mental state, which latter was so pronounced, that many of her friends said "she was not right in her mind." These grave constitutional indications, accompanied, as they were, by intense local pain, made me suspect that there might be more than an inflammation of the ovaries, possibly a malignant growth. The patient stated that her first attack of serious illness was fifteen years previously, and it commenced with a severe pain in the left side of the pelvis, which gradually increased year by year, extending to the right side. Frequently the pain was "so sharp, that she screamed with agony." She had consulted many physicians, and had had a great variety of treatment; fly-blisters were repeatedly applied to the lower part of the abdomen; for nine months she was treated for "inflammation and misplacement of the uterus," pessaries were used, and they only made her worse. Subsequently she was treated for "ulceration," and five years for "uterine congestion," with no better results. The next physician, after attending her for some time, said he could do nothing more, and relieved her distress by hypodermic injections of morphia. Her last physician treated her for valvular disease of the heart; said also, "the uterus was misplaced, and bound down by adhesions." He attempted to introduce pessaries, which, as before, "gave great distress."

For some time I continued the patient under observation, and could not see how local or general treatment would cure or benefit her. As far as I could judge, the ovaries were hopelessly diseased, and all her abnormal nerve symptoms were due to reflex irritation from these organs. I so informed the patient, and told her that their removal might be necessary. Nevertheless, I commenced with a course of treatment, hoping, if possible, to save her from the necessity of an operation; but there was no improvement. At last, after consultation, it was decided to remove the diseased organs, which, indeed, for this patient, seemed to be the only safe procedure. The operation took place on June 25, 1888. I was kindly assisted by Prof. Gill Wylie and Dr. Charles N. D. Jones. The patient made a rapid recovery, and soon was greatly improved in her general health, and in her mental and nervous ailments.

Though the patient had peritonitis and salpingitis, yet to me the most important consideration was the condition of the ovaries, since I suspected that this, probably, was the principal cause of her sufferings. The ovaries were found to be soft, flabby, and greatly enlarged, but on superficial observation there was no special evidence of disease. Morbid changes cannot always be recognized by merely surveying an organ externally, or slicing it in various directions; even cancer, in its early stages, cannot by this method be determined. Many profound pathological changes can be certainly known only by microscopical examination. I studied these ovaries microscopically in Dr. C. H. Heitzman's laboratory, and saw, for the first time, the elongated, branching, protoplasmic bodies, afterwards recognized as endothelia in a morbid change, surrounded by numerous blood-vessels containing blood corpuscles. Dr. Heitzman, with his vast experience as a pathologist, acknowledges that he had never before seen anything of the kind, as he remarked, "it gives a startling appearance"; and in August, 1885, when I was making drawings of these bodies, the written diagnosis then made under his direction was: "Net-celled sarcoma, starting from corpus luteum of the left ovary." I continued the study of this growth, and I find another drawing, made during the same month, with the written diagnosis: "Bundles of fibrous connective tissue, breaking down into sarcoma elements." Finally, we concluded that it was "an aveolar sarcoma, or a malignant form of endothelioma;" and Dr. Heitzman thought possibly the patient would not survive a year, since a large portion of the normal structure of the ovary was already destroyed and replaced by this apparently malignant growth.[1]

The second patient, in whom I recognized the same peculiar formation, was a young woman, brought to me by her mother on March 30, 1887. She was pale, cadaverous, emaciated, and cachectic, and looked as if she was far advanced in phthisis. With such grave constitutional symptoms, I was surprised to find no disease of the lungs, or organic derangement of any of the vital organs. I found a passably good condition of all the organs and organic functions, and no special trouble, except in the ovaries, which were greatly enlarged, extremely sensitive, and had a doughy,

1. It is pictured, and the case reported, in the *Medical Record*, August 22, 1886.

soft touch, which one might have diagnosticated as due to forming abscesses, except they were both alike affected, and had been in this state a long period. The patient said she had for years suffered with constant pain and distress on each side of the pelvis, and great tenderness in the regions of the ovaries. There was evidently some unusual form of disease in these organs, which was seriously impairing her general health. The more I saw the patient, the more I became convinced of this, and especially that "overmuch treatment" did not, in the least, benefit her. She had been under the care of a number of physicians and specialists, and had had long continued "local treatment," but without favorable results. As far as I could judge, nothing would restore the patient to health, or give her a chance of prolonged life, but the removal of the diseased organs, which "were enlarged into tumors," and were evidently the seat of some form of degeneration. I informed the husband of the state of affairs. He was anxious that the operation should be performed, gave his written consent, and laparatomy was performed in May, 1887. The patient made a rapid recovery, and I have reason to believe that by the operation her life was saved.

During the Spring and the following Summer I studied in the laboratory sections from these ovaries, made drawings of the same, and saw conclusively that every constituent part of the ovary was breaking down into peculiar branching protoplasmic bodies, accompanied by the formation of an infinite number of red blood-corpuscles and blood-vessels. I repeatedly assured myself of this fact, and in May, 1887, I presented before the New York Pathological Society, a specimen from this growth, calling attention to the new formation of blood-corpuscles and blood-vessels, to the crowding of many of the endothelia with granular matter and hematoplasts, the juvenile forms of the red blood-corpuscles.

At my request, Prof. Prudden, at that time President of the New York Pathological Society, took a piece from one of the ovaries, had sections of the same prepared, and mounted in his laboratory, returning me a slide, on which was written the diagnosis, "carcinoma."

I sent a slide prepared from the same ovaries to Prof. Waldeyer, of Berlin. He replied, October 18, 1887, that it was "carcinoma;" and certainly the symptoms did seem to point to the existence of this disease. Still, the new growth in the ovary, treated according to what I considered the best method, viz., first hardening in half of one per cent. solution of chromic acid, and after staining with ammoniacal carmine, mounting in pure glycerine, showed clearly the forming and ready formed blood-corpuscles and blood-vessels. This finding, of necessity, would exclude the diagnosis, "carcinoma," as the constituent epithelia of cancer could never produce, or be accompanied by a new formation of blood-corpuscles and blood-vessels.

I returned repeatedly to the study of this strange formation; studied it in different ovaries, and its symptoms in different individuals. It was, and is to this time, continually a new revelation, and I believe there is yet much more to be found out in regard to it, and that it has a significance that we, as yet, do not understand. I said in the article above referred to: "When we look at this rapidly growing formation and the great mass of granules, the impression forces itself upon us that it may be malignant. Further investigation may prove it so. Why this growth should have the power of destroying every structure of the ovary, why an ovary should so degenerate, or what is its pathological significance? are still questions for consideration. Certain it is, that this formation is as surely accompanied with manifestations of ill-health as is the breaking down of lung tissue; and the symptoms of the two diseases are, in many respects, similar.

Even in December, 1891, when examining in one case the peculiar shaped corpuscles, I called Dr. Heitzman's attention to them, and he remarked: "It resembles spindle-celled sarcoma." Dr. Francis Foerster remarked at the Academy of Medicine, May, 1892, that the growth was "destructive and progressive."

Dr. H. C. Coe says, in the July number of the *American Journal of Medical Science*, "All agree regarding the histological peculiarities and its close resemblance, anatomically at least, to a malignant neoplasm."

I am convinced that endothelioma in the ovaries is as serious as is pus in the tubes, and equally demands an operation. Before I knew there was such a disease as "endothelioma," I recognized in every single instance that the ovaries were profoundly affected, and sufficiently altered to demand extirpation; that the disease was more serious than oöphoritis, that it was a form of degeneration that was destroying the health of the individual, and endangering her life.

Dr. Coe further says in the same journal:

It is highly desirable to know, not only if it is possible to recognize at the examining-table an ovary which is the seat of endothelioma, but also if it gives rise to symptoms different from those of ordinary chronic oöphoritis, which form a more urgent indication for prompt removal by laparatomy.

In many instances, at least, the disease can be recognized at the examining-table. Endothelioma being always accompanied by acute and sub-acute inflammation, necessarily, the symptoms must be more marked than those of ordinary chronic oöphoritis.

An interesting feature that I observed for the first time in the ovaries of the second patient mentioned above, was that the *ova* were diseased. This possibly was the real cause of the woman's sterility, though she had, in addition, interstitial and suppurative salpingitis, either of which would have been sufficient to produce an incurable sterility; with ruined *ova*, however, there was no chance of conception.

Usually, where there is sufficient disease to call for the extirpation of an organ, it is also sufficient to destroy its physiological functions. It is not the operation that makes the woman sterile; *it* only removes a cause of danger and of suffering.

Another marked instance of endothelioma I had in the Spring of 1887. The patient had the same pallor, emaciation, and intense local pain. She exhibited symptoms of consumption, and, in the opinion of her friends, was going into rapid decline. Her constitution was so broken, and her general health so enfeebled, that she was no longer able to work. The patient was sent me by a physician of Bridgeport, who had given her much local treatment, and who wrote: "Everything possible has been done, and evidently an operation is demanded." The patient entered the Woman's Hospital of Brooklyn. She lay, day after day, suffering from this extreme pain and feebleness, looking so cadaverous and emaciated, that one would have supposed she had phthisis; but the lungs were comparatively healthy, as were all other organs except the ovaries, and these gave indication of some profound, but obscure derangement.

After consultation it was decided to remove the diseased organs, and thereby preserve the patient's strength and vital resources, to contend with, as was supposed, an incipient lung trouble. The operation was performed on May 13, 1887. The patient made an excellent recovery, was able to leave the hospital on the 24th. She gained in strength, health, and flesh, had no longer any threatened lung trouble, and was soon able to resume her former occupation. She wrote a few months after: "It seems strange to be free from pain." And two years later, on June 14, 1890, she wrote: "I am in perfect health after fifteen years of suffering." In January, 1892, the patient was still in good health and able to work.

Almost the entire ovary of this patient was found filled with the growth under consideration; it extended to the periphery, and was gradually invading and replacing every constituent tissue. Hematoblasts were present in abundance within the endothelioma, and the granular matter could be seen shaping itself into blood-corpuscles. In some fields of the microscope were seen the gradually forming blood-vessels, while in other places could be seen lakes of blood, without any confining walls.

The tissues around this growth are invariably found in a state of intense inflammation. The growth is preceded by oöphoritis, and produces it. This breaking down of the normal tissue of the ovary would account for patient's suffering, her great prostration, and her continued feebleness. In studying this and other cases, I saw, repeatedly, that endothelioma was not only accompanied by local pain, pallor, progressive emaciation, but also by certain nervous disturbances, and generally by profuse monorrhagia. The ovaries are usually found to be large, sensitive, and prolapsed.

In 1884, Mrs. G., 38 years of age, consulted me for continued distress and intense pain in the pelvis. She had the same symptoms, not so strongly pronounced, nor did I recognize their significance. Her menstruation was so profuse that, at first, the existence of a uterine myoma was suspected, but, finally, I diagnosed pelvic inflammation and endometritis. Treatment did not seem to improve her condition, so I requested Prof. B. F. Dawson to examine the patient. He recognized that there was pelvic and uterine disease, and advised a continuation of the treatment. This was done; still the improvement was not satisfactory. Subsequently, Dr. C. C. Lee, Surgeon of the New York State Woman's Hospital, kindly consented to see the patient. He also considered with me the question of an operation, but we finally decided to continue treatment. There were no better results; so that, at the end of a few months, I advised the patient to return to her home and desist from farther medical attendance.

In 1889, this patient again called on me; five years of suffering and still growing worse; five years more of married life and still sterile. I again took her history, and at this time, from her symptoms as above mentioned, and without hesitation, I pronounced the disease to be endothelioma. It was afterwards found that the endothelioma had changed to angioma and hematoma. The right ovary was enlarged to a blood cyst the size of an orange, and bound by dense adhesions to the wall of the pelvis. Thus the disease had advanced to a more dangerous state, and the patient's strength was more exhaused, and her sufferings were so great she had become an opium eater. She now urged that something be done. But even at this time, and after having witnessed the disease in so many instances, yet the pallor, the weakness, and profound constitutional disturbance of this patient made me, for months, hesitate and delay, until finally recognizing that the case was growing more and more critical, I decided, even with her unfavorable conditions, to perform the operation. It was done on February 16, 1891, with the kind assistance of Dr. R. T. Morris, Dr. Currier, and Dr. C. N. D. Jones. As a further test of the diagnosis, I said, before beginning the operation: "On the right there is an endothelioma changing to hematoma; on the left, I hope the disease is not so far advanced; and we may feel justified in leaving the uterine appendages on that side."

No sooner had I attempted, by the gentlest manipulations, to remove the enlarged ovary on the right side, than it ruptured, quantities of blood escaping into the peritoneal cavity. The ovary was transformed into a hematoma, and a portion of the wall was so exceedingly thin that it ruptured from the slightest touch. The appendages on the left were diseased, and all the surgeons present agreed that they should be removed. The patient made an excellent recovery, and in two weeks was able to return to her home in a neighboring city.

This case proves again that endothelioma may be diagnosticated from the symptoms; that continued treatment will not cure it, and that the disease steadily progresses and becomes more and more dangerous. Further, I believe that if the uterine appendages in this patient had been removed five years previously, it would have insured to her years of more vigorous health. Leaving them resulted in no good, they were of no service, they were doing positive injury, and, finally, they placed the patient's life in peril.

In 1888, Mrs. C. was brought to me from Bridgeport, Conn., by her physician. The patient was then 26 years of age, married several years, without children. She complained of such constant distress in the pelvis, that she was compelled to walk bent over, and was not able to do her work or attend to her household duties.

Though the patient looked sick, yet at first I suggested to her physician that she have further treatment. This was to give the woman a possible chance of bearing children. It seemed so extremely sad for one so young and lately married to be deprived of all hope of the sacred privilege of motherhood; yet, upon examination, I found conditions that demanded immediate and more radical measures. There was pyosalpinx of the tubes, on the right there was a pus cavity, and I had good reason to believe that a large portion of each ovary was destroyed by an endotheliomatous growth, and that the latter, probably, was the special cause of her acute pain. The patient insisted upon immediate relief, and her physician likewise advised laparatomy. She was admitted into the Woman's Hospital of Brooklyn. The incurability of the diseased organs was still further demonstrated, and after some constitutional treatment, oöphorectomy was performed. The conditions were found as previously diagnosticated, and the ovaries contained, in addition to endothelium, gyromatous formations.

Gyroma, as the name indicates, is a convoluted mass, and in full development is a homogenous, firm structure, frequently occupying the whole area of the section of the ovary, replacing almost all the normal anatomical elements. It usually results from morbid changes in the structureless membrane of a ruptured Graafian follicle, but may also arise from certain changes in the arteries, such as endarteritis obliterans or waxy degeneration of the same. The first time I recognized this formation was in 1887, when I was making microscopical researches of certain diseased ovaries, and on September 9, 1887, I presented the same to the New York Pathological Society as formations of dense fibrous connective tissue, or, "fibromata." The patient, in whose ovaries gyroma was first recognized, applied at the out-door department of the Woman's Hospital on April 23, 1887.

She was feeble, emaciated, cachectic, and had all the appearances of phthisis; but from an examination of the lungs, this could be excluded. Indeed, I had to eliminate disease from every organ except the ovaries, and these I found in a state of intense inflammation, one being enlarged to the size of a small orange, and adherent. The patient was extremely nervous, in an abnormal mental state, and complained principally of distress in the pelvis. She had been married eleven years, and had given birth to seven or eight children. When admitted into the hospital she was not able to sit up, was put to bed, and suffered with constant pelvic pain, so great that she begged again and again to be relieved by an operation, and seemed grieved and dissatisfied at every delay. We regarded the patient as tuberculous, and would not then consider the operation, because of the apparently poor prospect of the patient surviving. Besides, we could not see, at that time, how the removal of the diseased ovaries could so radically benefit her general conditions, or restore to health one so seriously affected. As she continued to grow worse and her symptoms became more alarming, we decided to perform the operation, in hopes of relieving her, at least, in this respect, and that, possibly, relief of the more grave symptoms might follow. The operation took place on May 21, 1887, and proved to be one of great difficulty. The left ovary was enlarged into a blood cyst, and ruptured on removal; the neighboring tissues were tender, and had a tendency to bleed,—even the fundus uteri oozed blood on the slightest touch. The broad ligaments tore like wet paper, tore from the cornu of the uterus, and melted under the secured ligature, so that at one time the whole left broad ligament was unfolded, countless vessels pouring forth their life-current, and in a moment the pelvis was welling up full of blood. The bleeding points were quickly secured, the clots removed, and the peritoneal cavity flushed out with an abundance of warm water, sterilized by heat.

The patient made an excellent recovery, was relieved of pain, gained flesh and strength, looked well, all appearance of phthisis disappeared, her mind seemed stronger, her mental functions were normal, and, in short, the results of the operation were in every way beyond our expectations. Her husband said she had not been so well for fifteen years. She commenced at once her heavy labors, doing the household work and washing for a family of eight persons. The gyroma, angioma, and endothelioma, with the accompanying oöphoritis, no doubt, caused in this patient the sickness, suffering, and constitutional derangements.

A more marked instance of gyromatous formations was found in a patient who consulted me in August, 1888:

There were the same symptoms: anemia, pallor, emaciation, local pain, and more or less anomalous manifestation of the nervous system. She was 49 years old, mother of eight children. The uterus was fixed on the right by inflammatory adhesions, the result of repeated attacks of peritonitis, which probably, as in the preceding case, had been induced by sepsis at her last confinement. A few years previously the patient had been told by an eminent specialist in Philadelphia, that she had inflammation of the ovaries. Since, her sufferings had continued to increase and her condition to grow worse, till her nervous system was broken down and in an exceedingly irritable state. For two months I gave her local and constitutional treatment, without any visible improvement. After the removal of the diseased organs, which had caused the local distress as well as the reflex irritation, the patient at once began to gain in health and strength, and to show an improved mental and physical condition.

In the report of this case to the New York Pathological Society, in 1888, I said: "Right ovary much enlarged, and most of it occupied by a fibroid growth, while in other portions of the ovaries there was a number of small, nodular fibromata."

The nodules were what we now understand by the name of gyroma, and, like endothelioma, are the result of an inflammation, and probably both are produced by some infection, as sepsis at confinement, or from pyosalpinx.

Though gyroma is frequently accompanied by endothelioma, yet, as I said in an article published in the *New York Journal*, May 10 and 17, 1890, "It exists in many cases where there is no trace of endothelioma." Gyroma is an entity in itself, and, as such, may appear waxy, pigmented, or reduced to embryonal tissue; eventually, however, it changes to endothelioma. Further investigation has proved that gyroma is frequently the first stage of endothelioma. Though there is a similarity of symptoms, yet I have thought that in gyroma the nervous and mental disturbances are more pronounced than in endothelioma. I have never seen a case of gyroma in which the nervous and mental conditions were not to some extent morbid. Formations of such firmness and density must necessarily cause not only local distress, but serious reflex irritations; and they are, probably, the principal cause of ovarian hysteria.

A young woman, to whom I was called a few years ago in Ansonia, Conn., was confined to her bed, weighing but seventy pounds, nervous, hysterical, at times insane, and more than once had threatened to take her life. She was carried in her husband's arms to the Woman's Hospital, and while there had hallucinations that could not be dispelled. The uterine appendages were found to be enlarged, sensitive, and low down behind a retroverted uterus. Every evacuation of the bowels caused extreme pain and almost a death-like weakness; menstruation was accompanied by suffering, and a prostration so extreme that it did not seem possible for her feeble frame long to endure it. The patient had pyosalpinx on both sides, and pelvic peritonitis. There seemed to be no other cure than the removal of the diseased organs.

After consultation, it was decided to perform the operation, which was done in July, 1887. Dr. A. N. Jacobus was present, and assisted. The patient made a good recovery; at the end of the second week, went up and down stairs without assistance, and rode out; at the end of the fourth week, she accompanied her husband home, and that day walked as much as a mile. Subsequently her general health and mental state still more improved, and she was soon able to attend to her household duties. The ovaries, when examined microscopically, were found not only to be in a state of intense oöphoritis, but there were large gyromatous formations; which latter, no doubt, were the cause of, or certainly intensified, the morbid conditions of the nervous system.

If such disturbances accompany and are symptoms of gyroma, should these symptoms be found, it is logical to infer that gyroma exists. It thus became an interesting question to me to ascertain whether or not, in some of my former patients who had marked hysteria, hysterical insanity, or hystero-epilepsy, if, besides oöphoritis and salpingitis, gyromatous formations were not also present. I commenced immediately to make investigations in this direction:

Miss C., a patient who was remarkably hysterical, morbid in her mental manifestations, and had more than once declared that she was going to kill herself and all her family, was brought to me by her parents in June, 1884. She had been suffering for many years, and, according to her own statement, had been attended at intervals by "thirty different physicians." In hopes of doing her good, and, if possible, curing her without resorting to an operation, I treated her continuously for four months; yet, at the expiration of this time, she was as sick as before, still had pain, and still was profoundly hysterical. I invited Prof. B. F. Dawson, and, subsequently, Prof. Gill Wylie, to see the patient in consultation. Both examined her to find out if something more could not be done for her relief in the way of palliative measures. Both decided that an operation was demanded. The father urged that it be done, and was disappointed at my delaying another month. It took place on October 15, 1884. Dr. Wylie, Dr. J. H. H. Burge, and Dr. C. N. D. Jones were present, and kindly assisted. Ether was administered by Dr. John Merrit. The patient made a good recovery, gradually improved physically and mentally.

Soon after the operation, I studied, with the microscope, the pathological specimens, and the diagnosis was subacute oöphoritis; but now, referring again to the same slides, I found, besides the focci of intense inflammation, large extensive gyromatous growths, which I then noted, but regarded as obscure pathological changes. I find, also, by referring to my book of drawings, that I had then, in 1885, drawn large gyromata without understanding what they were or knowing their significance. The written diagnosis at the time was: "Remnants of corpus luteum, inflamed myxomatous tissue, and tortuous arteries in consequence of pressure." This patient had also pus tubes, the infection from which probably caused the oöphoritis and gyroma.

Another case of hysterical insanity, upon which, for unmanageable disease of the uterine appendages, I operated in January, 1887, subsequently studied the morbid changes in the ovaries, and reported the same in the *American Journal of Obstetrics*, February, 1888, as "oöphoritis, etc." Now, from recollecting the symptoms of the patient, I surmised that she likewise had gyroma, reëxamined the slides, and found extensive formations of those growths, which would explain, I think, her peculiar nervous condition.

Another feature in the ovaries of the last-named patient were the diseased *ova.* The patient was only 22 years of age, yet not a single normal *ovum* was found in the cortex of either ovary. In one section I counted as many as forty-seven ova, all in a pathological state, the vesicula, the most vital part, being reduced to a waxy mass, deeply stained by the ammoniacal carmine.

If I had decided, in this case, to remove only the more diseased parts of the ovary, leaving a portion with the idea of giving the patient a chance of becoming a mother, I would have had a most disappointing result, and for the patient a second operation would have been necessary. In all the ovaries which I have removed, and all of them I subsequently studied microscopically, I do not know a single one where the person would have been benefitted by leaving a part of the organ *in situ.*

In 1883, I had a patient with hystero-epilepsy :

She was wildly hysterical, nervous, restless, sleepless, near the border land of insanity, had spasms often, as many as fifteen occurring during one night. Sometimes she was unconscious, again would spring up in bed, showing almost superhuman strength, or be doubled up in agonizing pain, the muscles being rigid, teeth clenched, and difficulty of breathing ; or, perhaps, she would crowd under the bed-clothes screaming with fright and trying to keep some imaginary being from killing her. For a long time these convulsions and tetaniform contractions were confined to the menstrual period, but later on she had every day most distressing spasms, jerking and twisting of the limbs, and horrible contortions of the body.

The patient first consulted me in October, 1882 ; 27 years old, nine years married, had never been pregnant. The uterus was found to be retroflexed and retroverted; on each side was a mass the size of an orange, fixed by inflammatory adhesions. She had been treated for years by bromides, blisters, Charcot's method, etc. So great had been her sufferings that she had become a slave to morphia ; her whole skin was riddled by the hypodermic needle. For several months I gave her local treatment in hopes of reducing the inflammation. Knowing then little of Tait's operation,[1] yet, independently and without thinking of this operation, I was impressed that these diseased masses should be

1. One of the first presentations of the subject in this country was made by Dr. T. A. Emmett at the New York Obstetrical Society, December, 1882, by exhibiting some specimens from Lawson Tait. The proceedings of this meeting were published in April, 1882. I first learned the subject in Tait's work, "Pathology and Treatment of Diseases of the Ovaries."

removed, that the uterine appendages could never be restored to health or normal activity, that they were only a source of distress to the system, and that the patient would be infinitely better without them. This seemed to me common sense and natural. For months I studied the patient's case, and became more and more convinced it was the only way to give relief or save life. At my request, Dr. Benjamin Westbrook saw the patient, and advised the trial of massage for one month. Prof. B. F. Dawson also saw the patient in consultation with me, and said that an operation should be performed without delay. Still I hesitated, put off from time to time even against the wishes of the patient and her friends, and thereby incurring their displeasure. The operation finally took place on the 12th day of the following May, Prof. Dawson very kindly performing the most of it. Dr. J. H. H. Burge, Dr. Frank Rockwell, and Dr. C. N. D. Jones assisting. The ovaries were found enlarged, the Fallopian tubes, two inches in diameter, were full of pus and adherent to and coiled sausage-like around the ovaries. Dr. Dawson said he had never seen so bad a case. He reported the operation and presented the specimens to the New York Obstetrical Society, also before his class at the Post-Graduate School.

Soon after the operation, I studied, with the microscope, the ovaries, and diagnosticated oöphoritis, etc. But now, recollecting the patient's peculiar nervous conditions, I was certain she must have had gyroma. Again examining the same microscopical slides, sure enough, there were immense gyromatous masses, and in a state of intense acute inflammation!

In a report of this case, in the *American Journal of Obstetrics*, November, 1884, I said: "The operation should have been performed in this patient two or three years before it was." A few weeks after the publication I received a letter from Mr. Lawson Tait, in which he says: "I agree with you absolutely. The only regret about all such cases is that they are allowed to go so long without an operation."

These hard, fibrous formations were, no doubt, the exciting cause of the patient's morbid, nervous symptoms. Is it any more strange, may I ask, that they, by reflex irritation, should excite serious neuroses than that reflex irritation from pathological ovaries should cause osteo-malacia? Fehling says: "Osteo-malacia is a reflex tropho-neurosis of the bones, dependent upon ovarian activity, and Thorn gives a case (*Centralblatt für Gynäkologie*, 1891, No. 41,) of a patient who had severe pains in the pelvic bones, with marked deformity, being unable to walk, and showing progressive emaciation and edema. The diseased uterine annexæ

were removed, after which the patient improved rapidly, the pains in the bones disappeared, she was soon able to walk, and a year after the operation was perfectly well. The "severe pains" and "progressive emaciation" lead one to suppose that patient also had endothelioma of the ovaries?

I wish here to emphasize that however alarming the nervous and mental symptoms may be, or however serious the general constitutional condition, yet the operation of oöphorectomy should not for a moment be considered, unless there is unmistakable proof that the ovaries are themselves seriously and irreparably diseased, and in such a state as to destroy the health, comfort, and comparative usefulness of the individual. This I have stated several times, and in an article published nearly two years ago, I said:

> I denounce the removal of the uterine appendages for any cause, neurotic conditions, constitutional disturbance, or any reason, except for incurable disease of the organs themselves; and I would not advise an operation in every case, even if there is incurable disease of the named organs.[1]

Some authors have reported good results from performing this operation for mental and neurotic ailments, still I cannot conceive that the removal of healthy ovaries will ever cure any neurosis. Normal organs can never cause abnormal symptoms, nor can their removal in any way result beneficially. As the *Medical Record*, of January 8, 1892, says: "Operations under these circumstances are founded upon equally irrational and absurd principles."

Gyroma of the ovary is a disease, and must necessarily excite abnormal nerve manifestation, by pressure, irritation, and destruction of nerve fibers, producing local distress as well as reflex neuroses. One patient who, at times, was almost insane from suffering, the condition of the ovaries caused continued and intense pain. Subsequently, I found, besides the general oöphoritis, that the Graaffian follicles were surrounded by layers of fibrous, hyaline, and inflamed cartilaginous tissues; and in these dense structures were imprisoned and compressed delicate non-medullated nerve fibers, which would explain the patient's neuralgia. This case is reported, and the imprisoned nerve fibers pictured, in an article published in the *American Journal of Obstetrics*, February, 1888. It is the first time, to my knowledge, that nerve fibers have been traced in inflamed ovarian tissue, at least, with such clearness as this specimen exhibited. The patient, after the

1. *New York Medical Journal*, May 10 and 17, 1890.

operation, was relieved and restored to health. That the nervous symptoms disappear, after the removal of such hard formations, proves that they are the cause of suffering, and of morbid, neurotic, and mental manifestations.

Gyromatous formations are illustrated in the *New York Medical Journal* of May 10 and 17, 1890. Such hard masses must produce discomfort and bodily derangements, and are probably the cause of many obscure mental and nervous disorders. In 1886, I visited La Salpétriére, in Paris, and had not only the pleasure of hearing Charcot's clinical lectures, but repeatedly walked through the immense old wards, with their hundreds of sick women, and had an opportunity of studying a number of those cases in connection with their history. Many of them gave evidence of extreme mental and bodily suffering, women whose lives were wrecked mentally and physically. I query now if gyroma in the ovaries of these sick women would not account for many of their bodily sufferings, and the abnormal functions of the brain and nervous system. Their position in life exposed them to the chances of infection.

If compressed nerve fibers are in connection with the vaso-motor system, epileptic fits may result. No doubt there are a few cases of epilepsy due to ovarian irritation; or, in a few, ovarian irritation may cause epilepsy; and this comparatively few may possibly be helped or cured by the operation of oöphorectomy. Out of a number of cases of epilepsy, or hystero-epilepsy, I have in three instances performed oöphorectomy, not, however, for the epilepsy, but because I considered that the ovaries or uterine appendages were past cure, and doing serious injury to the individual, and possibly by reflex irritation might have caused or, at least, aggravated the epilepsy. It is remarkable that each of three cases of epilepsy, which were really operated for ovarian disease, was found to have gyroma. As long as these patients continued under observation they showed marked improvement; and, probably, by continued care there might have been to each an entire restoration to health. This is exceedingly satisfactory, especially when we remember that epilepsy results from such obscure causes, and that when patients have to depend upon bromide, the Lord have mercy upon them, for a surfeit of salts for months and years often produces no good results.

Here naturally arises the question in regard to the assertion, frequently made, that oöphorectomy may cause, and in some instances has produced, insanity. I have never seen or known of an instance; but I do recognize the fact that any great shock to the system, from surgical operation or otherwise, may disturb the mental equilibrium, the same as puerperal insanity sometimes follows difficult parturition. Kiernan (*Medical Standard*, Vol. X., No. 3,) has found 146 cases of profound mental change following operations; among them thirty-five cases after operation for cataracts. These acute types of mental affection following operations are aptly diagnosticated as "acute confusional insanity." I had one patient, a woman of remarkable intelligence, who was temporarily insane after the removal of the mammary gland for cancer, but I have never had an instance of insanity after the removal of the uterine appendages; on the contrary, I have known a number of patients whose mental conditions were greatly improved after, or, rather, in consequence of this operation.

It is surprising that these gyroma formations have so long been regarded as normal, and called corpora lutea spuria in involution, and supposed to be the cicatrization that follows the rupture of a Graaffian follicle. Leopold has finely painted plates, showing the large typical corpus luteum of a recently ruptured follicle. It is pictured the same way in all the books and has been almost universally accepted. Careful microscopical investigation proves that these bodies are the results of disease. In an article published in the *New York Medical Journal*, May 10 and 17, 1890, I assert: "What observers have termed corpora lutea spuria are evidently nothing else than anomalous menstrual bodies (gyroma) and endothelioma, changing to angioma and hematoma."

I have repeatedly traced the commencement of the morbid change, seen the follicular membrane become the seat and center of an intense inflammation, gradually growing thicker, firmer, broader, and more and more solid; inflammatory corpuscles change to extremely dense fibrous connective tissue, in which a waxy or colloid basis substance is deposited, and thus the membrane grows to a great convoluted mass, eventually spreading out to large proportions, sometimes occupying the area of a whole section of the ovary, or it may develop into a number of round, nodular, fibromata of various sizes. Often I have seen inflammation in one part doing its work of destruction while other portions of the membrane were perfectly normal. I have traced the inflammation gradually progressing, changing more and more this originally insignificant membrane till it became immensely increased in size, a hard, solid formation, irritating the surrounding structures, or itself changing to endothelioma, and, finally, forming a dangerous blood cyst. The follicular wall is originally a fine, delicate membrane of a high refraction, and, when normal, is thrown into graceful folds and found imbedded in the ovarian stroma, and could not of itself be a source of irritation or of any disturbance in any way. I have found it thus unchanged in great numbers in the ovary of women past seventy years of age. Not one of them had formed a gyroma, the supposed corpus luteum spurium, though the women had menstruated regularly for thirty or forty years, and may have had several children. If there had been oöphoritis or salpingitis, we would have found the supposed corpora lutea vera.

Dalton says: "The vascular membrane takes the increased development by which it becomes thickened and convoluted, and tends to fill the cavity of the follicle." This describes exactly the abnormal changes that result from inflammation. He says further: "On cutting it open, the corpus lutem is seen to consist of a central coagulum and a convoluted wall." This describes gyroma changing to endothelioma. Such processes have been described and recognized by many authors as normal, whereas they are in reality the results of morbid processes, the outcome of oöphoritis and salpingitis.

163 DeKalb Avenue.

